# Multiplex Detection of Immunoglobulins Uncovers Intrathecal IgA Elevation in Multiple Sclerosis

**DOI:** 10.3390/cells15100870

**Published:** 2026-05-10

**Authors:** Leonard Apeltsin, Sakthi Asokan, Brendan Freitas, Krish Karekar, Samuel Guzman, Enrique Alvarez, Xiaoli Yu

**Affiliations:** 1 Anomaly Insights, New York, NY 10018, USA; 2 Department of Neurology, University of Colorado Anschutz Medical Campus, Aurora, CO 80045, USA

**Keywords:** multiple sclerosis, cerebrospinal fluid, intrathecal immunoglobulin synthesis, IgA, IgM, IgG subclasses, antibody isotypes, Luminex multiplex assay, biomarker

## Abstract

**Background**: Intrathecal IgG synthesis is a well-established hallmark of multiple sclerosis (MS), yet the roles of other immunoglobulin isotypes remain under investigated. This study aimed to comprehensively profile immunoglobulin distributions in cerebrospinal fluid (CSF) and plasma from MS patients and neurological controls. **Methods**: Paired CSF and plasma samples from 23 MS patients and 20 neurological controls were analyzed using a multiplex Luminex-based immunoassay targeting IgG1–4, IgA, and IgM. The findings were validated using commercial ELISA kits and Western blot analysis. **Results**: Multiplex analysis revealed a selective intrathecal enrichment of IgA and IgM in MS CSF, with significantly higher levels compared to both matched plasma and control CSF. IgG1 was modestly elevated in MS CSF by ELISA but not by multiplex assay. Western blotting confirmed a robust elevation of IgA in MS CSF, providing qualitative support for intrathecal enrichment rather than definitive proof of synthesis. **Conclusions**: These findings uncover previously underappreciated selective enrichment of IgA and IgM in the MS CNS compartment, complementing, rather than replacing, the established IgG-centric paradigm. The results suggest alternative antibody-mediated mechanisms in MS and highlight the importance of assay selection in biomarker discovery, which suggests IgA as an emerging component of MS immunopathology.

## 1. Introduction

Multiple sclerosis (MS) is a chronic inflammatory disease of the central nervous system (CNS) characterized by demyelination and neurodegeneration. A defining immunological hallmark of MS is the presence of oligoclonal bands (OCBs) composed of immunoglobulin G (IgG) in the cerebrospinal fluid (CSF), detected in over 90% of patients [1]. These bands are predominantly of the IgG1 subtype, with IgG3 OCBs found in approximately two-thirds of cases; IgG2 and IgG4 OCBs are comparatively rare [2,3]. Additionally, Elevated CSF IgG1 concentrations have been consistently reported in MS [4,5,6], and CSF IgG levels show a strong correlation with those in serum [3]. Beyond the CNS, aggregates enriched in IgG1 and IgG3 have been identified in peripheral blood, which caused complement-dependent neuronal cytotoxicity and may serve as diagnostic markers to distinguish MS patients from healthy controls [6,7,8]. Our recent study demonstrated that MS CSF IgG aggregates contribute to OCBs and causes neuronal cytotoxicity [9]. Together, these findings point to a persistent, compartmentalized B cell response within the CNS. In addition to IgG, intrathecal IgM synthesis is frequently observed in MS, with IgM OCBs associated with a more aggressive disease course [10,11]. This suggests that multiple antibody isotypes contribute to MS immunopathology from its earliest stages.

In contrast, the potential role of immunoglobulin A (IgA) in MS has long been understudied [12]. Unlike IgG and IgM, IgA has not historically been considered a clinically useful diagnostic or prognostic marker, and quantitative IgA analysis is not routinely performed in MS evaluations [13]. Earlier surveys reported that only ~18% of MS patients show any evidence of intrathecal IgA synthesis, a frequency far lower than that of IgG or even IgM [13,14]. Furthermore, the very low concentration of IgA observed within the CSF has hampered the establishment of qualitative and quantitative analyses [13,15]. Earlier studies that did look for IgA in MS found it often clinically insignificant: for example, one 1985 analysis detected oligoclonal IgA bands in some MS patients’ CSF but noted these IgA bands did not correlate with the clinical course or stage of MS [16]. The low IgA detection rates originated from low-sensitivity assays that underestimate polymeric IgA, not biological irrelevance. As a result, research attention focused on IgG and IgM, rendering IgA a “forgotten” isotype in MS for decades.

Recent work, however, suggests that IgA responses are active within the MS CNS and may influence disease processes. Postmortem studies of MS brain tissue have found IgA-producing plasma cells in inflamed areas and IgA bound to damaged nerve fibers, suggesting that IgA or IgA-containing immune complexes may contribute to axonal injury [17]. Furthermore, a pivotal study by Pröbstel et al. [18] revealed that during active MS relapses, patients exhibit elevated levels of IgA specificity within the CSF. This was accompanied by a higher CSF-to-serum IgA ratio, a key indicator of intrathecal production of the antibody within the CNS itself, rather than simple leakage from the blood. Significantly, the study demonstrated that this increase might be due to the migration of IgA-producing B cells from the gut to the inflamed CNS, suggesting that these cells may play a nuanced role in the neuroinflammatory process. The most recent report showed that Ocrelizumab therapy in MS partially spared memory IgA B cells, providing further evidence of the complex roles of IgA and IgA B cells [19]. Table 1 lists the studies of IgA in MS (Table 1).

Recent advances in detection methods have also challenged the long-held assumption that IgA plays a negligible role in MS. Ultrasensitive isoelectric focusing assays now reveal intrathecal IgA in a substantially higher proportion of MS patients than previously recognized, with IgA oligoclonal bands detected in 43% of cases compared to only 17% of controls [13]. This discrepancy suggests that earlier failures to identify IgA involvement may have been due to methodological limitations rather than biological insignificance. Currently, no standardized approach exists for quantifying intrathecal IgA, and techniques vary widely, from nephelometry and ELISA to isoelectric focusing, leading to inconsistent results [12]. Compounding this issue, IgA exists in both monomeric and polymeric forms, and standard assays like ELISA have been shown to underestimate dimeric IgA levels [20,21,22].

The present study leverages multiplex bead-based immunoassays, ELISA, and Western blotting to systematically evaluate IgA, IgM, and IgG subclass distributions in paired CSF and plasma samples from MS patients and controls. By comparing these techniques, we aim to clarify whether IgA enrichment in the MS CNS is a consistent feature detectable across independent methods or an artifact of assay-specific biases. Our findings not only reinforce the emerging paradigm of IgA involvement in MS but also underscore the critical influence of methodological approaches on the interpretation of immune responses in neuroinflammatory disease. The selective enrichment of intrathecal IgA in MS suggests its role in disease pathogenesis, providing additional support for the idea that gut-derived IgA+ B cells may act as systemic immune regulators in MS. Rather than replacing the IgG paradigm, our findings suggest that IgA represents an underrecognized and potentially complementary immunoglobulin response in MS that warrants further investigation in larger cohorts.

## 2. Materials and Methods

### 2.1. Plasma and Cerebrospinal Fluid Samples

With the approval of the University of Colorado Institutional Review Board (COMIRB #00–688, #13-3007), plasma and cerebrospinal fluid from MS and control patients with other central nervous system disorders were collected at the University of Colorado Hospital. Plasmas were collected after centrifugation of blood samples at 2000× *g* for 10 min; CSFs were immediately centrifuged at 500× *g* for 10 min, and the supernatant was collected. Both CSF and plasma were stored at −80 °C until use. Patient demographics, including age and sex, as well as treatment status, are listed in Table 2. 

### 2.2. Multiplex Immunoglobulin Isotype Quantification Detecting IgG1-4, IgA, and IgM

Quantification of immunoglobulin isotypes and subclasses in paired plasma and CSF samples was performed with a bead-based multiplex fluorescence assay using the Bio-Plex Pro Human Isotyping Panel (Bio-Rad, 171A3100M, Hercules, CA, USA). This 6-plex panel is a premixed multiplex kit for detecting human immunoglobulins IgG1, IgG2, IgG3, IgG4, IgA, and IgM within each sample. The assay utilizes a mixture of magnetic beads conjugated to various antibodies. Each bead type acts independently of the others and functions similarly to a sandwich ELISA with a fluorescent reporter rather than an HRP tag. This kit contains a bead mixture capable of detecting IgG1-4, IgA, and IgM. MS and control plasma, as well as paired CSF, were diluted 1:5 and 1:1500, respectively, in the kit diluent buffer. Prior to each step, the plate and beads were washed with a kit wash buffer using a Bio-Plex Pro wash station (Bio-Rad, 30034376). All incubations were performed in the dark, at room temperature, while shaking at 850 RPM. Samples and standards were incubated with antibody-conjugated magnetic beads in a 96-well plate for 1 h. After sample incubation, the bead-Ig complexes were incubated with biotinylated detection antibodies for 30 min. The biotinylated sandwich complexes were then incubated with streptavidin-phycoerythrin for 10 min. The completed complexes were finally resuspended in the provided assay buffer. Fluorescence intensity at 562 nm was measured using a Bio-Plex 200 system (Bio-Rad, 171000205). According to manufacturer specifications, Bio-Plex Pro™ Human Isotyping assays demonstrate intra-assay coefficients of variation (CVs) typically ≤10% and inter-assay CVs ≤ 15%, supporting reliable quantification of low-abundance immunoglobulins in biological fluids.

### 2.3. Human IgG Subclass ELISA

IgG subclasses were quantified by commercial Human IgG Subclass ELISA Kit (Thermo Fisher Catalog #99-1000, Waltham, MA, USA) following recommended protocol. Plasma samples were thawed and diluted with Diluent Buffer to ratios of 1:4500 for IgG1 detection, 1:3000 for IgG2 detection, and 1:1500 for IgG3 detection. CSF samples were thawed and diluted with Diluent Buffer to ratios of 1:15 for IgG1 detection, 1:10 for IgG2 detection, and 1:5 for IgG3 detection. Human IgG Subclass Standard was reconstituted, and serial dilutions were made with Diluent Buffer. 50 μL of the appropriate human IgG subclass-specific antibody was added to the appropriate wells. Then, 50 μL of diluted samples and standards was added to the respective wells. The plate was covered and incubated at room temperature on an orbital shaker for 30 min. After 30 min, the contents were removed by inverting the plate into the sink. 300 μL of diluted Wash Buffer was added to all wells. The plate was placed on the orbital shaker for 15 to 30 s, and then the contents were removed by inverting the plate into the sink. Wash steps were repeated three more times.

The Peroxidase Anti-Human IgG conjugate was diluted according to the kit’s instructions at a 1:50 ratio with Diluent Buffer. 100 μL of diluted Peroxidase Anti-Human IgG conjugate was added to all wells. The plate was covered and incubated at room temperature on an orbital shaker for 30 min. After 30 min, the contents were removed by inverting the plate into the sink. 300 μL of diluted Wash Buffer was added to all wells. The plate was placed on the orbital shaker for 15 to 30 s, and then the contents were removed by inverting the plate into the sink. Wash steps were repeated three more times. 100 μL of ready-to-use TMB solution was added to each well. The plate was protected from light and left to incubate at room temperature for 30 to 50 min on the orbital shaker. 100 μL of ready-to-use Stop Solution was added to each well. The absorbance of the samples at 450 nm (OD_450_) was measured using a microplate reader. A 6-point standard curve was generated, and then concentrations were calculated using a log-log fit.

### 2.4. Human IgA ELISA

#### ELISA Detection of IgA in CSF and Paired Plasma

Quantification of IgA in paired plasma and CSF samples was achieved using a commercially available sandwich ELISA kit (Immunology Consultant Laboratory, E-80A). Plasma and CSF were diluted 1:20,000 and 1:67, respectively, with the kit diluent buffer. Standards were prepared according to the instructions of the certificate of analysis. Pre-coated strips were incubated with 100 μL of diluted sample or standard at room temperature for 30 min before being washed 4 times with kit wash buffer. Strips were then incubated with 100 μL of HRP-conjugated detection antibody at room temperature for 30 min. The wash was repeated to remove excess HRP before the addition of 100 µL of TMB substrate. TMB was incubated at room temperature, in the dark, for 10 min before the reaction was stopped with 100 μL of stop solution. Wells were immediately measured for absorbance at 450 nm using a BioTek Synergy 2 plate reader (BioTek, 7131000, Winooski, VT 05404, United States) running Gen5 1.11.5 software (BioTek, 7130202).

### 2.5. Western Blots Detecting IgA in Paired CSF and Plasma

Neat CSF and paired plasma, diluted 1:300 in TBS (Bio-Rad, 1706435), were each mixed at a 1:1 ratio with 2x-Laemmli buffer (Bio-Rad, 1610737) with 5% β-mercaptoethanol (Sigma, M6250100ML, St. Louis, MO 63103, USA). All samples were denatured in boiling water for 8 min, then cooled on ice for a minimum of 5 min. SDS-PAGE was performed using 4–20% Mini-PROTEAN TGX gels (Bio-Rad, 4561096) and 1× Tris-Glycine/SDS buffer (Bio-Rad, 1610734). Gels were loaded with 10 uL of sample and electrophoresed at 160 V for 115 min. Protein was transferred to 0.45 µm (PVDF) membranes (Bio-Rad, 1620262) at 100 V for 60 min in 1× Tris-Glycine buffer (Bio-Rad, 1610734) with 20% methanol. Membranes were blocked in 1× casein (Vector, SP-5020, Newark, CA 94560, United States) for 45 min at room temperature. Blots were probed overnight at 4 °C with goat anti-human IgA antibody (1:5000, Bio-Rad, STAR141). Membranes were incubated for 1 h at room temperature with HRP-conjugated donkey anti-goat antibody (1:5000, Jackson ImmunoResearch, 705035003, West Grove, PA 19390, USA). Detection was performed using the Super-Signal West Femto Maximum Sensitivity Substrate (ThermoFisher, 34094, Rockford, IL, USA). Blots were imaged using the iBright FL1500 Imaging System (ThermoFisher, A44241) and quantified with ImageJ software (1.54P).

## 3. Results

### 3.1. Multiplex Luminex Profiling Identifies Selective Enrichment of IgA and IgM in MS CSF

**Figure 1:**
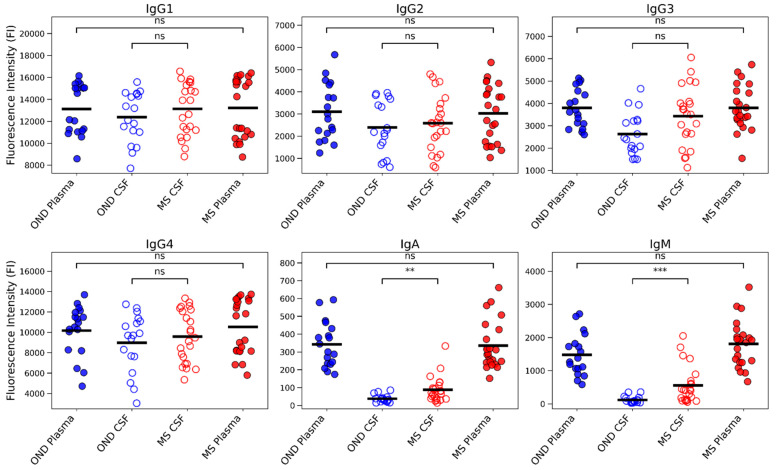
Multiplex Luminex measurement of immunoglobulin isotype levels in MS and OND plasma and CSF. High-throughput multiplex Luminex profiling of paired samples shows levels of each immunoglobulin (Ig) isotype, expressed as fluorescence intensity (FI). No significant differences were detected for any IgG subclass in either compartment. In contrast, CSF IgA (** *p* < 0.01) and IgM (*** *p* < 0.001) were elevated in MS relative to OND, indicating selective enrichment of these isotypes in the CNS compartment.

High-throughput multiplex Luminex analysis of paired CSF and plasma samples revealed a selective elevation of IgA and IgM in the MS CNS compartment (Figure 1). Across all IgG subclasses, no significant differences were observed between MS and other neurological disease (OND) controls in either CSF or plasma. In contrast, CSF IgA was significantly higher in MS compared to OND (*p* < 0.01), and CSF IgM was elevated to an even greater extent (*p* < 0.001). These differences were compartment-specific, as plasma levels for both isotypes were comparable between groups. The findings indicate that IgA and IgM are selectively enriched intrathecally in MS.

### 3.2. CSF/Plasma Ratios Point to Intrathecal Synthesis of IgA and IgM in MS

**Figure 2:**
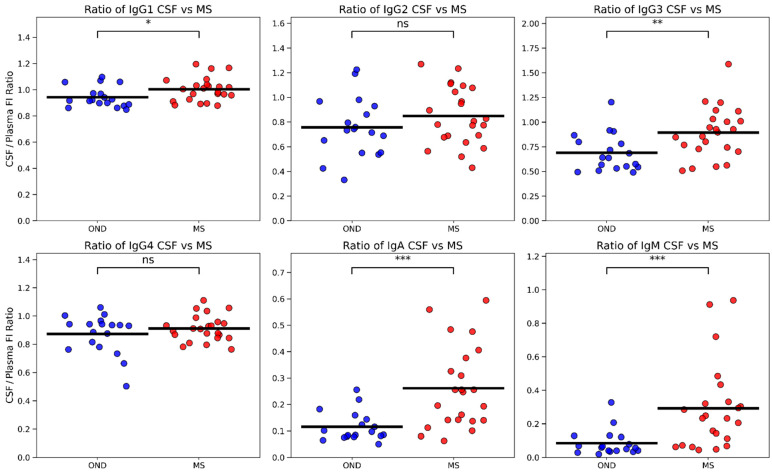
CSF/plasma immunoglobulin ratios reveal increased IgA intrathecal synthesis in MS. Ratios of CSF to plasma Ig levels, derived from multiplex Luminex measurements, highlight marked increases for IgA and IgM (*** *p* < 0.001) in MS compared to OND, supportive of intrathecal enrichment consistent. IgG3 ratios were also significantly elevated (** *p* < 0.01), whereas IgG1 showed a modest increase (* *p* < 0.05), and IgG2 and IgG4 ratios were unchanged.

To contextualize compartmental differences, we computed CSF-to-plasma ratios from the Luminex measurements (Figure 2). IgA and IgM ratios were markedly increased in MS relative to OND (*p* < 0.001), consistent with intrathecal enrichment but not definitive evidence of local synthesis. Among IgG subclasses, only IgG3 showed a significant ratio increase (*p* < 0.01), with IgG1 displaying a modest elevation (*p* < 0.05) and no changes for IgG2 or IgG4. These results reinforce a prominent intrathecal signal for IgA and IgM. CSF/plasma ratios are indirect metrics and may be influenced by blood–CSF barrier permeability and assay-specific sensitivity.

### 3.3. Singleplex ELISA Fails to Detect IgA Differences

**Figure 3:**
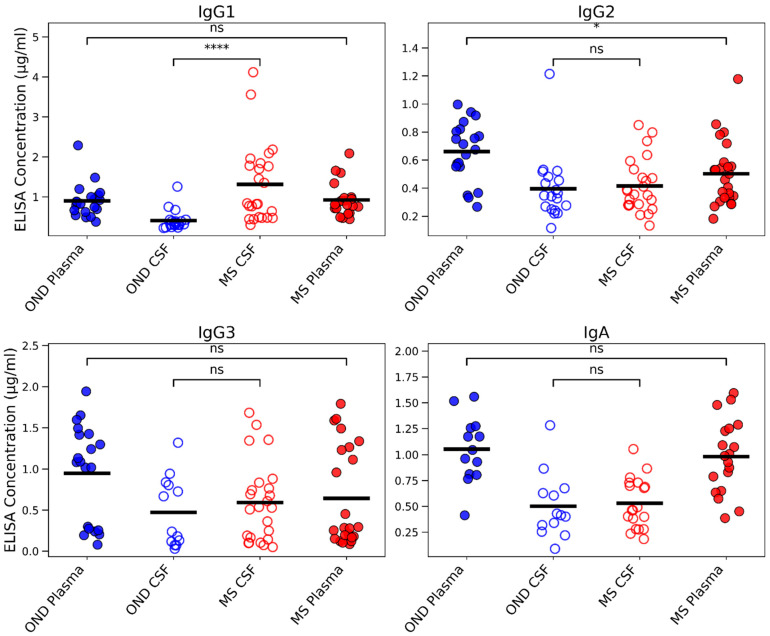
Singleplex ELISA measurement of immunoglobulin isotype concentrations in MS and OND plasma and CSF. Individual concentrations (µg/mL) of IgG1, IgG2, IgG3, and IgA were determined by singleplex ELISA in paired plasma and CSF samples. IgG1, IgG2, and IgG3 were selected for analysis because these subclasses have higher effector functions. IgG1 was markedly elevated in MS CSF compared to OND CSF (**** *p* < 0.0001), and IgG2 showed a modest increase in MS plasma (* *p* < 0.05). No significant differences were observed for IgG3 or IgA. These ELISA-derived concentration profiles differ from the multiplex Luminex results in Figure 1, where IgA was selectively enriched in MS CSF. The discrepancy between platforms may stem in part from the known limitations of ELISA in detecting dimeric IgA, as polymeric forms are often underestimated in this assay. The divergence also highlights that different analytical platforms can yield distinct immunoglobulin signatures in MS, potentially reflecting disease-related immunoglobulin aggregation. In solution-phase multiplex Luminex, epitopes may be more exposed, influencing detection patterns.

To compare these multiplex findings with conventional singleplex immunoassays, concentrations of IgG1, IgG2, IgG3, and IgA were measured by ELISA in the same paired CSF and plasma samples (Figure 3). ELISA detected a robust increase in IgG1 in MS CSF versus OND (*p* < 0.0001), along with a modest rise in IgG2 in MS plasma (*p* < 0.05), but no significant changes in IgG3 or IgA in either compartment. Thus, ELISA did not reproduce the IgA elevation observed by Luminex. This discrepancy may stem from ELISA’s known limitations in detecting dimeric IgA, as this assay often underestimates polymeric forms. Unlike ELISA, which relies on protein immobilization on a solid phase where conformational masking or steric hindrance may occur, Luminex operates in the solution phase and can better detect epitopes exposed on polymeric IgA. Beyond methodological differences, these contrasting results suggest that different analytical platforms may reveal distinct immunoglobulin signatures in MS, potentially reflecting disease-related aggregation. The solution-phase nature of Luminex likely enhances detection sensitivity by minimizing steric hindrance, making it more effective at identifying aggregated immunoglobulins. The discrepancy between Luminex and ELISA likely reflects methodological differences between solution-phase and solid-phase assays rather than assay failure. While reduced ELISA sensitivity for polymeric/dimeric IgA is supported by prior literature, no direct assessment of IgA polymeric state was performed in this study; therefore, all mechanistic explanations are presented as hypotheses rather than conclusions.

### 3.4. Western Blot Confirms CSF IgA Enrichment in MS

**Figure 4:**
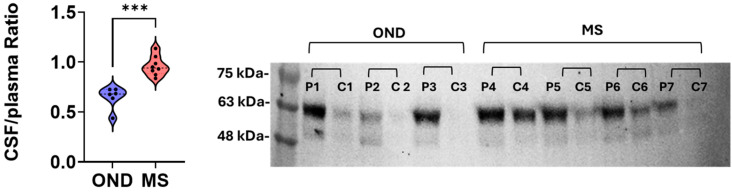
Western blot detecting IgA in CSF and paired plasma. Neat CSF and paired plasma (1:300 dilution) were separated on 4–20% SDS-PAGE followed by blotting with PVDF membrane. IgA was detected with goat anti-human IgA, followed by incubation with secondary antibody Donkey anti-goat IgG-HRP and chemiluminescent substrate. OND: Other neurological disorders. OND = 6; MS = 8. Mann–Whitney test, *** *p* = 0.0007.

To independently validate the IgA signal, we performed Western blotting on paired CSF and plasma from MS and OND cohorts (Figure 4). IgA bands were readily detected, and the CSF signal was significantly higher in MS than in OND (*p* < 0.001). These findings confirm elevated intrathecal IgA in MS and underscore the value of multi-method validation for immunoglobulin profiling. Western blotting was performed on a subset of samples (MS = 8, OND = 6). This subset is underpowered for quantitative validation and serves as qualitative, confirmatory evidence of elevated IgA signal rather than definitive validation of intrathecal synthesis.

## 4. Discussion

Our study challenges the long-held view that IgA plays a minor role in MS by demonstrating its clear enrichment in the CSF of patients when measured with highly sensitive techniques. These findings do not negate the central role of IgG in MS but instead expand the immunoglobulin landscape of the disease. Using multiplex magnet bead-based assays, we detected intrathecal increases in both IgA and IgM, a finding that conventional ELISA missed but was confirmed by Western blotting. This discrepancy underscores how assay design influences the detection of immunoglobulins, particularly those in low abundance or aggregated forms.

Our findings align with recent studies demonstrating that IgA responses in the MS CNS are more prevalent than previously recognized, especially when detected using high-sensitivity or solution-phase methods. The discrepancy between our Luminex and ELISA results likely stems from both biological and technical factors. Biologically, IgA exists in monomeric and polymeric forms, with polymeric IgA potentially enriched under inflammatory conditions [23]. In MS CSF, IgA may also form disease-specific aggregates, similar to those of IgG aggregates reported in peripheral blood [6,8]. Technically, ELISA’s reliance on immobilized antigens can obscure epitopes on polymeric or aggregated IgA, reducing sensitivity. Generally, plate-based ELISAs are known to underestimate aggregated protein concentrations [24], whereas Luminex’s solution-phase format minimizes steric hindrance and better preserves conformational epitopes. Consistent with this, a prior study found that a multiplex Luminex assay detected significantly higher IgA levels than traditional methods [25], suggesting that our Luminex data captured additional IgA (likely polymeric or sterically hindered forms) that ELISA missed. Notably, Western blot validation confirmed that elevated IgA in MS reflects a genuine biological phenomenon, rather than an artifact.

The selective enrichment of IgA and IgM, without proportional increases in most IgG subclasses, points to unique B cell activation patterns in the MS CNS. While intrathecal IgM has been linked to aggressive disease, the role of IgA remains unclear. One possibility is that IgA-producing cells migrate from mucosal sites such as the gut into the CNS during neuroinflammation, as proposed by Pröbstel’s group [18]. This hypothesis raises important questions about whether mucosal immune modulation could influence CNS disease activity.

IgA is the most abundant immunoglobulin at mucosal surfaces and plays a critical role in immune exclusion and maintaining homeostasis [26]. While traditionally considered protective, emerging evidence suggests that IgA can also contribute to autoimmune pathology under certain conditions. Monomeric IgA (mostly in serum) is less likely to form immune complexes.

Polymeric IgA (especially dimeric forms in the mucosa) can form larger immune complexes, particularly when glycosylation is abnormal [27]. In autoimmune diseases, such as IgA nephropathy (IgAN), systemic lupus erythematosus (SLE), and rheumatoid arthritis (RA), IgA immune complexes can deposit in tissues, driving inflammation by activating the complement system [28]. Although polymeric IgA represents a biologically plausible contributor to the enhanced signal detected by solution-phase multiplex assays, this interpretation remains speculative and is based on prior reports of assay-dependent IgA detection rather than direct molecular characterization in this study. Altered IgA glycosylation can promote immune complex formation and pathogenic inflammation [29], glycosylation status was not examined in this study and therefore remains a speculative contributor requiring future investigation. Further investigation into the glycosylation patterns of IgA and its potential contribution to immunocomplexes or Ig aggregates may shed light on its pathological roles in MS. Elevated IgA in MS CSF may reflect systemic mucosal immune activation, possibly originating from the gut. Our data support the previous study that B cells may secrete IgA locally in the CNS, contributing to inflammation or immune modulation [18]. Our assays do not distinguish secretory from serum IgA nor assess IgA glycosylation status. Our findings also highlight a broader challenge in MS research: methodological differences can obscure biologically meaningful signals. We acknowledge the limited cohort size and statistical power as a key limitation for the study; we further acknowledge that no clinical correlation was performed. Therefore, findings should be considered hypothesis-generating and require validation in larger, clinically stratified cohorts. Future studies should also investigate whether IgA levels correlate with clinical outcomes such as relapse rate or disability progression and whether they define distinct patient subgroups with unique pathophysiology.

## 5. Conclusions

Our findings demonstrate that IgA is selectively enriched in the CSF of MS patients when measured with sensitive techniques, challenging its historical dismissal as irrelevant. The discrepancy between multiplex assays (which detect elevated IgA/IgM) and ELISA highlights how methodological choices influence biomarker discovery. This work positions IgA as a potential player in MS neuroinflammation, suggesting that future studies should clarify its origins, clinical relevance, and therapeutic implications. We propose IgA as an emerging and complementary component of MS immunopathology rather than a replacement for the well-established IgG framework.

## Figures and Tables

**Table 1:** Studies on Intrathecal IgA in Multiple Sclerosis.

Study	Methodology	Key Findings	Implications
Sindic et al., 1984 [14]	Isoelectric focusing of CSF	Approximately 18% of MS patients exhibited intrathecal IgA synthesis.	Early evidence of IgA in MS is present but considered clinically insignificant.
Grimaldi et al., 1985 [16]	Oligoclonal band detection	IgA bands were detected in some MS CSF samples, but no correlation was found with disease stage.	IgA was dismissed as non-contributory to MS progression.
Zhang et al., 2005 [17]	Postmortem brain tissue analysis	IgA+ plasma cells and IgA deposits on damaged axons.	Suggested IgA may contribute to axonal injury.
Pröbstel et al., 2020 [18]	CSF/serum IgA ratio, flow cytometry, single-cell RNA-seq	Identified gut-derived IgA+ B cells migrating to the CNS during MS relapse; enriched in active lesions.	Supports gut-CNS immune axis; IgA+ B cells may drive neuroinflammation and serve as therapeutic targets
Muñoz et al., 2022 [13]	Ultrasensitive isoelectric focusing (IEF)	IgA OCBs were detected in 43% of MS vs. 17% of controls.	Higher prevalence than previously recognized; IEF is more sensitive than ELISA
Kliushnikova et al., 2025 [12]	Review of biological and clinical data	Summarized IgA’s role, highlighted detection challenges.	Advocated for standardized IgA assays and further clinical relevance studies

**Table 2:** Patient information.

Diagnosis	Total Patients	Gender	Average Age	CSF OCB Status	Treatment Status
Headache/Migraine	18	Female: 13 Male: 5	41.76	Negative:15 N/A: 2 Positive: 1	Treatment Naïve: 18
Multiple Sclerosis	23	Female: 14, Male: 9	38.75	Positive: 17, N/A: 5, Negative: 1	Treatment naïve: 16 Fingolimod: 2 Dimethyl fumarate: 1 Glatiramer acetate: 1 Natalizumab: 1 Off DMT: 1 N/A: 1

For control (headache) and MS, the table lists the total number of patients, gender distribution, average age at visit, CSF OCB status distribution, and treatment status.

## Data Availability

Data supporting the findings of this study are available from the corresponding author upon reasonable request; restrictions apply due to human subject privacy considerations.

## References

[1] Deisenhammer F., Zetterberg H., Fitzner B., Zettl U.K. (2019). The cerebrospinal fluid in multiple sclerosis. Front. Immunol..

[2] Kennedy P.G.E., George W., Yu X. (2024). The elusive nature of the oligoclonal bands in multiple sclerosis. J. Neurol..

[3] Beseler C., Vollmer T., Graner M., Yu X. (2017). The complex relationship between oligoclonal bands, lymphocytes in the cerebrospinal fluid, and immunoglobulin G antibodies in multiple sclerosis: Indication of serum contribution. PLoS ONE.

[4] Kennedy P.G.E., Graner M.W., Fringuello A., Zhou W., Pointon T., Alquatli K., Bisel S., Langford D., Yu X. (2022). Higher Levels of IgG3 Antibodies in Serum, But Not in CSF, Distinguish Multiple Sclerosis From Other Neurological Disorders. J. Neuroimmune Pharmacol..

[5] Kennedy P.G.E., Graner M., Pointon T., Li X., Tanimoto K., Dennison K., Im G., Fringuello A., Zhou W., Graner A. (2022). Aberrant Immunoglobulin G Glycosylation in Multiple Sclerosis. J. Neuroimmune Pharmacol..

[6] Zhou W., Graner M., Beseler C., Domashevich T., Selva S., Webster G., Ledreux A., Zizzo Z., Lundt M., Alvarez E. (2023). Plasma IgG aggregates as biomarkers for multiple sclerosis. Clin. Immunol..

[7] Apeltsin L., Xiaoli Y. (2025). IgG Biomarkers in Multiple Sclerosis: Deciphering Their Puzzling Protein A Connection. Biomolecules.

[8] Zhou W., Graner M., Paucek P., Beseler C., Boisen M., Bubak A., Asturias F., George W., Graner A., Ormond D. (2023). Multiple sclerosis plasma IgG aggregates induce complement-dependent neuronal apoptosis. Cell Death Dis..

[9] Asokan S., Zhou W., Fringuello A., Pointon T., Kennedy A., Freitas B., Tumas French J., Zhao H., Coughlan C., Alvarez E. (2026). CNS-compartmentalized IgG aggregates and glycosylation in multiple sclerosis contribute to oligoclonal bands and neuronal cytotoxicity. Front. Immunol..

[10] Oechtering J., Schaedelin S., Benkert P., Müller S., Achtnichts L., Vehoff J., Disanto G., Findling O., Fischer-Barnicol B., Orleth A. (2021). Intrathecal immunoglobulin M synthesis is an independent biomarker for higher disease activity and severity in multiple sclerosis. Ann. Neurol..

[11] Villar L.M., Masjuan J., González-Porqué P., Plaza J., Sádaba M.C., Roldán E., Bootello A., Alvarez-Cermeño J.C. (2003). Intrathecal IgM synthesis is a prognostic factor in multiple sclerosis. Ann. Neurol..

[12] Kliushnikova D., Otto F., Pilz G., Wipfler P., Harrer A. (2025). Intrathecal Immunoglobulin A Synthesis in Multiple Sclerosis: From Biological Aspects to Clinical Relevance. Biomolecules.

[13] Muñoz Ú., Sebal C., Escudero E., Sánchez M.I.G., Urcelay E., Jayo A., Arroyo R., García-Martínez M.A., Álvarez-Lafuente R., Sádaba M.C. (2022). High prevalence of intrathecal IgA synthesis in multiple sclerosis patients. Sci. Rep..

[14] Sindic C.J.M., Delacroix D., Vaerman J., Laterre E., Masson P. (1984). Study of IgA in the cerebrospinal fluid of neurological patients with special reference to size, subclass and local production. J. Neuroimmunol..

[15] Withold W., Wick M., Fateh-Moghadam A., Einhäupl K. (1994). Detection of oligoclonal IgA in cerebrospinal fluid samples by an isoelectric focusing procedure. J. Neurol..

[16] Grimaldi L.M.E., Roos R.P., Nalefski E.A., Arnason B.G. (1985). Oligoclonal IgA bands in multiple sclerosis and subacute sclerosing panencephalitis. Neurology.

[17] Zhang Y., Da R.-R., Hilgenberg L.G., Tourtellotte W.W., Sobel R.A., Smith M.A., Olek M., Nagra R., Sudhir G., Noort S.v.D. (2005). Clonal expansion of IgA-positive plasma cells and axon-reactive antibodies in MS lesions. J. Neuroimmunol..

[18] Pröbstel A.-K., Zhou X., Baumann R., Wischnewski S., Kutza M., Rojas O.L., Sellrie K., Bischof A., Kim K., Ramesh A. (2020). Gut microbiota–specific IgA+ B cells traffic to the CNS in active multiple sclerosis. Sci. Immunol..

[19] Garcia A., Rodriguez S., Dugast E., Lebrun-Frenay C., Thouvenot E., de Sèze J., Le Page E., Vukusic S., Doghri I., Berger E. (2025). Durable B-Cell Impairment While Sparing IgA B Cells After Ocrelizumab Therapy in Multiple Sclerosis. Ann. Clin. Transl. Neurol..

[20] De Fijter J.W., van den Wall Bake A., Braam C., van Es L., Daha M. (1995). Immunoglobulin A subclass measurement in serum and saliva: Sensitivity of detection of dimeric IgA2 in ELISA depends on the antibody used. J. Immunol. Methods.

[21] Delacroix D.L., Dehennin J.P., Vaerman J.P. (1982). Influence of molecular size of IgA on its immunoassay by various techniques. II. Solid-phase radioimmunoassays. J. Immunol. Methods.

[22] Dingess K.A., van Dam P., Zhu J., Mank M., Knipping K., Heck A.J., Stahl B. (2021). Optimization of a human milk–directed quantitative sIgA ELISA method substantiated by mass spectrometry. Anal. Bioanal. Chem..

[23] Hansen I.S., Baeten D.L.P., den Dunnen J. (2019). The inflammatory function of human IgA. Cell Mol. Life Sci..

[24] Janssen L., Sobott F., De Deyn P.P., Van Dam D. (2015). Signal loss due to oligomerization in ELISA analysis of amyloid-beta can be recovered by a novel sample pre-treatment method. MethodsX.

[25] Andersen N.J., Mondal T.K., Preissler M.T., Freed B.M., Stockinger S., Bell E., Druschel C., Louis G.M.B., Lawrence D.A. (2014). Detection of immunoglobulin isotypes from dried blood spots. J. Immunol. Methods.

[26] Li Y., Jin L., Chen T. (2020). The Effects of Secretory IgA in the Mucosal Immune System. Biomed. Res. Int..

[27] Ding L., Chen X., Cheng H., Zhang T., Li Z. (2022). Advances in IgA glycosylation and its correlation with diseases. Front. Chem..

[28] Maillard N., Wyatt R.J., Julian B.A., Kiryluk K., Gharavi A., Fremeaux-Bacchi V., Novak J. (2015). Current Understanding of the Role of Complement in IgA Nephropathy. J. Am. Soc. Nephrol..

[29] Suzuki H., Novak J. (2021). IgA glycosylation and immune complex formation in IgAN. Semin. Immunopathol..

